# Misoprostol Versus Oxytocin for Labor Induction in Term Prelabor Rupture of Membranes: A Systematic Review of Their Efficacy and Safety

**DOI:** 10.7759/cureus.95195

**Published:** 2025-10-22

**Authors:** Mona Sidahmed Hassan Abdalla, Mohamed Hamid El Hassan Hamid, Egbal Lutfi Mohamed Salih, Reem Babkir Altayeb Abdullah, Aya Sidahmed Hassan Abdalla, Tabareh Saifaldeen Abdulla Awadalkarem, Roaa Fathi Ali Ahmed, Fatima Babikir Awadalseed Basheer

**Affiliations:** 1 Obstetrics and Gynecology, Maternity and Children Hospital, Buraydah, SAU; 2 Obstetrics and Gynecology, Dorset County Hospital NHS Foundation Trust, Dorchester, GBR; 3 Obstetrics and Gynecology, Sabt Alaya Hospital, Alkhaldiyah, SAU; 4 Obstetrics and Gynecology, National Gaurd Hospital - Imam Abdulrahman Bin Faisal Hospital, Dammam, SAU; 5 Faculty of Medicine and Health Sciences, Nile Valley University, Atbara, SDN; 6 Obstetrics and Gynecology, King Fahad Hospital, Albaha, SAU; 7 Obstetrics and Gynecology, Ministry of Health, Nizwa, OMN

**Keywords:** efficacy, labor induction, misoprostol, oxytocin, safety, systematic review, term prelabor rupture of membranes

## Abstract

Prelabor rupture of membranes (PROM) at term is a common obstetric event requiring safe and effective labor induction. While oxytocin has traditionally been used for this purpose, misoprostol offers a potentially advantageous alternative due to its multiple routes of administration. This systematic review aims to compare the efficacy and safety of misoprostol versus oxytocin for labor induction in women with term PROM. A systematic search was conducted across PubMed, Embase, Scopus, and ClinicalTrials.gov for studies published in the last five years. Randomized controlled trials (RCTs) and comparative studies comparing misoprostol and oxytocin for labor induction in term PROM were included. Study selection, data extraction, and risk of bias assessment using the Cochrane Risk of Bias 2 (RoB 2) tool were performed by two independent reviewers. A narrative synthesis was conducted due to clinical heterogeneity.

Six studies were included. Efficacy-related findings were heterogeneous. Two studies found that oxytocin significantly reduced the time to delivery, particularly in nulliparous women with an unfavorable cervix. Conversely, two studies reported that sublingual misoprostol resulted in a shorter induction time and a significantly lower cesarean section rate. Other studies found no significant differences in vaginal delivery rates within 24 hours. Critically, both agents demonstrated comparable and reassuring safety profiles, with no significant differences in major maternal morbidities or adverse neonatal outcomes. Both misoprostol and oxytocin are effective and safe for labor induction in term PROM. The optimal choice is context-dependent; oxytocin may be preferable for faster delivery in monitored settings for specific subgroups, while sublingual misoprostol is a highly effective alternative that may reduce cesarean rates and offers logistical benefits. The choice should be individualized based on clinical setting, patient characteristics, and preferences.

## Introduction and background

Prelabor rupture of membranes (PROM) at term is a common obstetric condition, occurring in approximately 8-10% of pregnancies; it is defined as the spontaneous rupture of fetal membranes before the onset of labor at ≥37 weeks of gestation [1]. PROM presents a clinical challenge due to its association with maternal and neonatal complications, including chorioamnionitis, endometritis, postpartum hemorrhage, and neonatal sepsis [2]. Prompt and effective labor induction in women with term PROM is crucial to minimize these risks while promoting favorable maternal and perinatal outcomes [3].

Labor induction in term PROM can be achieved using pharmacological agents that stimulate uterine contractions [3]. Oxytocin, a naturally occurring peptide hormone, has long been the standard agent for labor induction due to its well-established efficacy in promoting coordinated uterine activity. However, its use necessitates continuous intravenous infusion, close fetal monitoring, and hospital-based oversight. Additionally, it carries potential risks, including uterine hyperstimulation and tachysystole, which can jeopardize fetal well-being [4].

Misoprostol, a synthetic prostaglandin E1 analogue, has emerged as a promising alternative for labor induction [5]. It offers several advantages, including multiple routes of administration (oral, sublingual, or vaginal), low cost, and dual action on cervical ripening and uterine contractions. Nevertheless, variations in dosage regimens, administration routes, and study designs have led to inconsistent findings regarding its comparative effectiveness and safety in term PROM [6]. While some studies have reported shorter induction-to-delivery intervals and higher vaginal delivery rates within 24 hours with misoprostol, others have raised concerns about uterine hyperstimulation and fetal distress [7].

Despite the growing number of trials and meta-analyses evaluating labor induction methods, a clear consensus has yet to be reached on the most effective and safest pharmacologic agent for managing term PROM. Clinical practices differ substantially between institutions and regions, with variability in protocol selection, misoprostol dosage, and oxytocin titration regimens. These inconsistencies underscore an ongoing evidence-based uncertainty regarding optimal induction strategies tailored specifically for PROM cases, where both maternal infection risk and induction efficiency must be carefully balanced. Hence, this systematic review aimed to synthesize and compare the current evidence on the efficacy and safety of misoprostol versus oxytocin for labor induction in term PROM. By consolidating recent data and evaluating key clinical outcomes, this review seeks to clarify the relative benefits and risks of each agent and to support the development of standardized, evidence-based induction protocols for women presenting with PROM at term.

## Review

Methodology

Eligibility Criteria

This systematic review included studies comparing misoprostol and oxytocin for labor induction in women with term PROM. To ensure the synthesis reflects the most up-to-date clinical evidence and current obstetric practices, only studies published within the past five years were considered. Randomized controlled trials (RCTs) and prospective comparative studies assessing efficacy and safety outcomes were eligible. Studies focusing on populations with preterm PROM, multiple gestations, or significant maternal or fetal comorbidities were excluded to ensure a homogeneous study population and reduce confounding factors.

Information Sources

A comprehensive literature search was conducted using four electronic databases: PubMed, Embase, Scopus, and ClinicalTrials.gov. These databases were selected to ensure broad coverage of both published and ongoing clinical research in the field of obstetrics. The search was limited to articles published in English in the last five years. Reference lists of included studies and relevant review articles were also screened to identify additional eligible studies.

Search Strategy

The search strategy combined controlled vocabulary (Medical Subject Headings (MeSH) terms) and free-text keywords related to labor induction, term PROM, misoprostol, and oxytocin. Boolean operators (“AND” and “OR”) were used to refine the search and capture all potentially relevant studies. The search strategy was tailored for each database to optimize sensitivity and specificity, ensuring the inclusion of all pertinent literature published within the defined timeframe. The detailed search strategy is presented in the Appendices section.

Selection Process

All retrieved records were imported into EndNote X9 for organization, and duplicates were identified and removed using the software. Two reviewers independently screened titles and abstracts against the eligibility criteria. Full texts of potentially relevant studies were then assessed for inclusion. Discrepancies were resolved through discussion and consensus, with a third reviewer consulted when necessary. This two-step selection process aimed to minimize selection bias and ensure comprehensive study inclusion.

Data Collection Process

Data from included studies were extracted independently by two reviewers using a standardized data extraction form. The form captured detailed information on study characteristics (author, publication year, country, and study design), participant demographics (age, parity, and gestational age at induction), intervention and comparator regimens (dosage, route, and timing), inclusion and exclusion criteria, and reported efficacy and safety outcomes. Any disagreements in data extraction were discussed between the two reviewers, and consensus was reached with the involvement of a third reviewer when necessary to ensure accuracy and consistency.

Data Items

Primary outcomes of interest were the induction-to-delivery interval, rate of vaginal delivery within 24 hours, and cervical ripening efficacy. Secondary outcomes included maternal adverse events such as uterine hyperstimulation, postpartum hemorrhage, and fever, as well as neonatal outcomes including Apgar scores, neonatal ICU admissions, and signs of fetal distress.

Risk of Bias Assessment

The methodological quality of the included RCTs was assessed using the Cochrane Risk of Bias 2 (RoB 2) tool [8]. This tool evaluates five domains: bias arising from the randomization process, deviations from intended interventions, missing outcome data, measurement of the outcome, and selection of the reported result. Two reviewers independently conducted the risk of bias assessment. Any differences in judgment were first discussed between the reviewers, and if agreement was not reached, a third reviewer was consulted to make the final decision, ensuring a transparent and reliable evaluation process.

Data Synthesis

A narrative synthesis was performed due to considerable heterogeneity across the included studies. Differences in study designs, participant characteristics, misoprostol dosing regimens, routes of administration, oxytocin infusion protocols, and outcome definitions limited the feasibility of performing a meta-analysis. Moreover, the variability in reporting statistical data (e.g., missing standard deviations, inconsistent measurement units) further restricted quantitative pooling. Therefore, descriptive synthesis was considered the most appropriate approach to summarize findings, allowing for meaningful comparison and interpretation of efficacy and safety outcomes across studies while emphasizing key trends and clinical relevance.

Reporting

This systematic review adheres to the PRISMA (Preferred Reporting Items for Systematic Reviews and Meta-Analyses) 2020 guidelines [9] for reporting systematic reviews, ensuring transparency, reproducibility, and methodological rigor in the identification, selection, and synthesis of evidence comparing misoprostol and oxytocin for labor induction in term PROM.

Results

Study Selection Process

**Figure 1:**
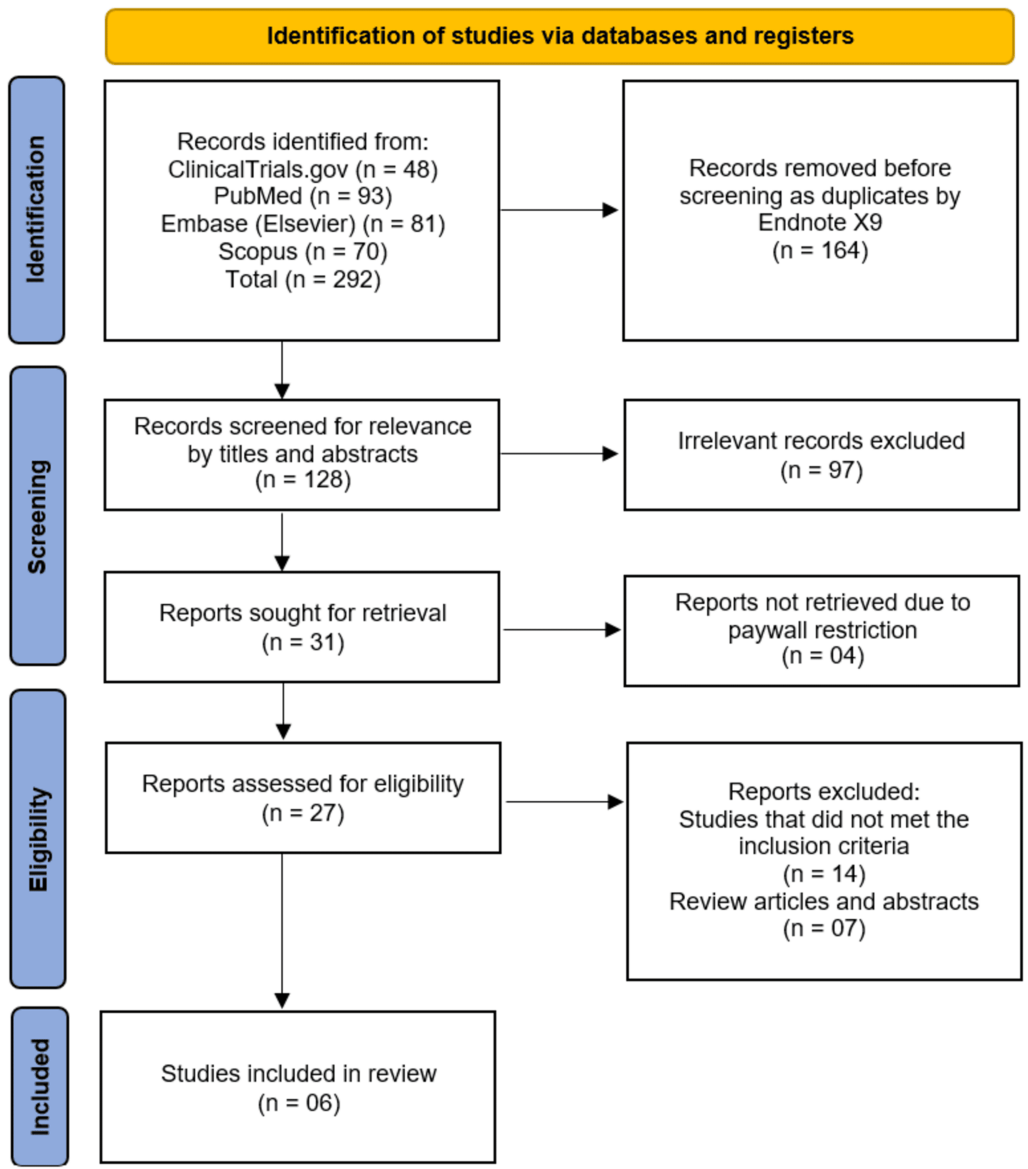
PRISMA flow diagram depicting the study selection process PRISMA: Preferred Reporting Items for Systematic Reviews and Meta-Analyses

The systematic search across four databases and registers (ClinicalTrials.gov, PubMed, Embase, and Scopus) initially identified 292 records. After the removal of 164 duplicate records, 128 unique studies were screened by their titles and abstracts. This screening process excluded 97 records that were deemed irrelevant to the review's objective. The full texts of the remaining 31 reports were sought for retrieval, of which four could not be accessed due to paywall restrictions. As a result, 27 full-text articles were assessed for eligibility. Out of these, 21 reports were excluded: 14 for failing to meet the predefined inclusion criteria and seven for being review articles or conference abstracts. Ultimately, this rigorous selection process led to the inclusion of six studies for qualitative synthesis in this systematic review (Figure 1) [10-15].

Study Characteristics

A total of six studies, comprising five RCTs [10-13,15] and one comparative clinical study [14], were included in this systematic review. The characteristics of these studies are summarized in Table 1. The studies were conducted across a range of countries, including the United States [10], Egypt [11,13,15], Thailand [12], and India [14], and were published between 2020 and 2024. The total sample size across all studies was 806 participants, with individual study sizes ranging from 55 to 200. All investigations focused on women with term PROM and compared various regimens of misoprostol (administered orally [10,11], sublingually [12,15], or vaginally [13]) to intravenous oxytocin. The primary outcomes reported consistently across the studies pertained to the efficacy of labor induction, most commonly measured as the induction-to-delivery interval or the rate of vaginal delivery within 24 hours, and the safety profile for both mother and neonate.

**Table 1:** Characteristics of tncluded studies RCT: randomized controlled trial; PROM: prelabor rupture of membranes; IOL: induction of labor; NICU: neonatal intensive care unit; PPH: postpartum hemorrhage; LSCS: lower segment cesarean section

Study	Country	Study design	Sample size (n)	Intervention (misoprostol regimen)	Comparator (oxytocin regimen)	Inclusion criteria	Primary outcomes reported
Bender et al. [10] (2024)	USA (urban tertiary care center, 2019-2023)	RCT	108 (52 misoprostol, 56 oxytocin)	Oral misoprostol	Intravenous oxytocin	Nulliparas ≥36 weeks gestation with PROM and unfavorable cervix (≤2 cm dilation and Bishop score <8)	Time from IOL to delivery (hours). Secondary: intraamniotic infection, cesarean delivery, composite maternal/neonatal morbidity, patient satisfaction
Ahmed et al. [11] (2023)	Egypt (Ain Shams University Maternity Hospital)	RCT	173	25 μg oral misoprostol tablet every 4, max 5 doses	Oxytocin infusion according to hospital protocol	Pregnant women with term PROM	Rate of vaginal delivery within 24 h (primary); secondary: time to active phase, induction-to-delivery interval, maternal pyrexia, nausea/vomiting, fetal distress, Apgar score, birth weight, NICU admission
Unthanan et al. [12] (2022)	Thailand	Randomized single-blind trial	170 (85 per arm)	Sublingual misoprostol	Intravenous oxytocin	Term pregnant women with PROM presenting for delivery	Induction time, duration of active phase, duration of second stage, cesarean section rate, intrapartum/maternal/neonatal complications, PPH, uterine rupture
Muhmed and Abdelmoaty [13] (2022)	Egypt	RCT	55	vaginal misoprostol	Intravenous oxytocin	Pregnant women with term PROM	Uterine tachysystole, hypersystole, efficacy, and safety of labor induction
Bhatu et al. [14] (2020)	India	Comparative clinical study	200 (oxytocin 100 and misoprostol 100)	Misoprostol	Oxytocin/syntocinon	Term pregnancies with PROM; both primipara and multipara	Vaginal delivery rate, LSCS rate, induction-to-delivery interval
Abdel-Aal et al. [15] (2020)	Egypt	Randomized single-blind trial	100	Sublingual 50 µg every 6 hours	Low-dose standard oxytocin infusion	Term singleton pregnancy with PROM and an unripe cervix	Duration of induction, duration of the second stage of labor, maternal side effects, and neonatal outcomes

Efficacy Outcomes

The efficacy of misoprostol versus oxytocin for labor induction in term PROM was evaluated through several key metrics, including the time from induction to delivery, rates of vaginal delivery, and cesarean section rates. The findings, however, were not uniform across the included studies.

Two studies reported a statistically significant advantage for oxytocin in reducing the time to delivery. Bender et al. [10] found that the median time to delivery was approximately 3.2 hours shorter in the oxytocin group compared to the oral misoprostol group, a difference that became more pronounced (four to five hours) in subgroups of patients with a BMI ≥30 or cervical dilation ≥1 cm. Conversely, two other studies demonstrated a significant benefit for misoprostol. Unthanan et al. [12] reported that the sublingual misoprostol group had a significantly shorter mean induction time (338 minutes vs. 399 minutes, MD: -61 min) and a lower cesarean section rate (13.3% vs. 28.8%, p=0.002). Similarly, Abdel-Aal et al. [15] observed that sublingual misoprostol resulted in a shorter duration of both the induction and the second stage of labor compared to oxytocin.

Other studies found no significant difference in the primary efficacy endpoints. Ahmed et al. [11] reported no significant difference in the rate of vaginal delivery within 24 hours between oral misoprostol and oxytocin (82.4% vs. 87.1%, p=0.394), although misoprostol was associated with shorter times to the active phase and delivery interval, particularly in multiparous women. Bhatu et al. [14] also found comparable overall efficacy, with misoprostol showing a slightly shorter induction-to-delivery interval in nulliparous women but similar times for multiparous women. A summary of these comparative efficacy and safety outcomes is provided in Table 2.

**Table 2:** Summary of efficacy and Safety Outcomes Across Studies BMI: body mass index; MD: mean difference; NS: not significant; RR: risk ratio; OR: odds ratio; CI: confidence interval; NICU: neonatal intensive care unit; NR: not reported

Study	Outcome	Misoprostol	Oxytocin	Comparative effect (RR/OR/MD with 95% CI, if reported)	Direction of effect	Safety notes (maternal/neonatal)
Bender et al. [10] (2024)	Time to delivery (overall longer with misoprostol; significant benefit for oxytocin in BMI ≥30 and cervix ≥1 cm), patient satisfaction is higher with oxytocin, no difference in cesarean, infection, or maternal/neonatal morbidity	Median delivery time: 18.1 h; mean satisfaction score: 26.3	Median delivery time: 14.9 h; mean satisfaction score: 29.0	MD ~3.2 h shorter with oxytocin (NS overall); 4-5 h shorter in subgroups (p=0.04); satisfaction +2.7 with oxytocin (p=0.03)	Favored oxytocin	No increased maternal or neonatal morbidity; safety comparable
Ahmed et al. [11] (2023)	Vaginal delivery within 24 h, time to active phase, induction-delivery interval, maternal pyrexia, nausea/vomiting, fetal distress, Apgar score, birth weight, NICU admission	Vaginal delivery within 24h: 82.4%; shorter induction-to-delivery interval	Vaginal delivery within 24h: 87.1%; longer induction-to-delivery interval	No significant difference in vaginal delivery (p=0.394); misoprostol shortened induction metrics	Misoprostol favored for induction speed; overall efficacy comparable	Both regimens are safe; no significant differences in maternal or neonatal adverse outcomes
Unthanan et al. [12] (2022)	Labor induction and delivery outcomes	Mean induction time: 338 min; cesarean rate: 13.3%	Mean induction time: 399 min; cesarean rate: 28.8%	Induction time MD: −61 min (significant), cesarean p=0.002	Favors misoprostol for induction time and cesarean	No significant maternal/neonatal complications; no postpartum hemorrhage or uterine rupture
Muhmed and Abdelmoaty [13] (2022)	Uterine tachysystole/hyperstole	Uterine tachysystole rate: 7.27; % of patients affected: 1.81%	Uterine tachysystole rate: 10.90; % of patients affected: 1.81%	NR	No significant difference	Maternal: uterine tachysystole/hyperstole
Bhatu et al. [14] (2020)	Labor induction efficacy, delivery outcomes, and induction-to-delivery interval	Induction-delivery interval (nullipara): 8.5 h; (multipara): 6.6 h	Induction-delivery interval (nullipara): 10.4 h; (multipara): 6.5 h	NR	Slightly favors misoprostol for nullipara; otherwise comparable	Misoprostol is safe at an appropriate dosage
Abdel-Aal et al. [15] (2020)	Induction and 2nd stage duration, maternal and neonatal outcomes	Shorter induction and 2nd stage duration; higher incidence of side effects	Longer induction and 2nd stage duration; lower incidence of side effects	NR	Favors misoprostol for labor duration; no difference for maternal/neonatal safety	Maternal: mild, non-significant side effects; neonatal: no difference

Safety and Maternal Outcomes

The safety profiles of both misoprostol and oxytocin were extensively evaluated, with a focus on maternal side effects and intrapartum complications. The majority of studies concluded that both agents were safe, with no significant differences in major maternal or neonatal morbidity.

Several studies specifically monitored uterine hyperstimulation. Muhmed and Abdelmoaty [13] found no significant difference in the rates of uterine tachysystole or hypersystole between vaginal misoprostol and oxytocin. Bender et al. [10] reported no increased risk of intraamniotic infection, cesarean delivery, or a composite measure of maternal morbidity. Similarly, Ahmed et al. [11] and Unthanan et al. [12] found no significant differences in adverse maternal outcomes such as pyrexia, nausea, vomiting, postpartum hemorrhage, or uterine rupture.

Regarding maternal satisfaction and side effects, the results were mixed. Bender et al. [10] found that patient satisfaction was significantly higher in the oxytocin group. Abdel-Aal et al. [15] noted that while maternal side effects were generally mild and non-significant, they were slightly more frequent in the sublingual misoprostol group, whereas the oxytocin group experienced fewer side effects.

Neonatal Outcomes

Neonatal outcomes were consistently reassuring across all six studies. Critical indicators such as Apgar scores, birth weight, incidence of fetal distress, and rates of admission to the NICU were comparable between the misoprostol and oxytocin groups [10-12,15]. None of the included studies reported any significant increase in neonatal morbidity or adverse outcomes associated with the use of either induction agent, affirming the neonatal safety of both misoprostol and oxytocin in the context of term PROM.

Risk of Bias Assessment

The methodological quality of the included studies was assessed using the Cochrane RoB 2. The majority of the trials were judged to have a low risk of bias. Specifically, five studies [10-13,15] demonstrated a low risk of bias across all domains, including the randomization process, deviations from intended interventions, missing outcome data, measurement of the outcome, and selection of the reported result. In contrast, one comparative clinical study [14] was assessed as having a high overall risk of bias. This judgement was primarily due to concerns in the randomization process, which was likely inadequate, and in the measurement of outcomes, where a lack of blinding posed a risk of detection bias. Therefore, the overall body of evidence is comprised predominantly of studies with a low risk of bias, with one exception (Table 3) [14].

**Table 3:** Quality assessment of included studies using the Cochrane RoB 2 tool

Study	D1: Randomization process	D2: Deviations from intended interventions	D3: Missing outcome data	D4: Measurement of the outcome	D5: Selection of the reported result	Overall risk of bias
Bender et al. [10] (2024)	Low	Low	Low	Low	Low	Low
Ahmed et al. [11] (2023)	Low	Low	Low	Low	Low	Low
Unthanan et al. [12] (2022)	Low	Low	Low	Low	Low	Low
Muhmed and Abdelmoaty [13] (2022)	Low	Low	Low	Low	Low	Low
Bhatu et al. [14] (2020)	High	Low	Low	High	Some concerns	High
Abdel-Aal et al. [15] (2020)	Low	Low	Low	Low	Low	Low

Discussion

This systematic review comprehensively evaluated the efficacy and safety of misoprostol versus oxytocin for labor induction in women with term PROM by synthesizing evidence from six contemporary studies [10-15]. The main finding of this analysis is that both agents are effective and generally safe options for this indication; however, they exhibit distinct efficacy profiles that may not be equivalent across all patient subgroups or clinical settings. The evidence suggests that while oxytocin may lead to a shorter time to delivery in certain populations, particularly nulliparous women with an unfavorable cervix, misoprostol - especially via the sublingual route - can be equally, if not more, effective in reducing the induction interval and lowering cesarean section rates in other contexts. Crucially, both medications demonstrated a reassuring and comparable safety profile for both the mother and neonate, with no consistent signal of increased serious adverse events.

The divergent findings regarding the primary efficacy outcome, the induction-to-delivery interval, underscore the complexity of comparing these pharmacologically distinct agents. The results from Bender et al. [10], which demonstrated a significant 3.2-hour reduction in delivery time with oxytocin, particularly in women with a BMI ≥30, present a compelling argument for its use in well-resourced, tertiary care settings where continuous electronic fetal monitoring is standard. This finding aligns with a large network meta-analysis by Alfirevic et al. [16], which concluded that oxytocin infusion was among the most effective agents for achieving vaginal delivery within 24 hours. The faster onset of action with intravenous oxytocin, providing immediate and titratable uterine stimulation, likely explains this temporal advantage in a closely monitored environment.

Conversely, the significant reduction in induction time and cesarean delivery rate observed with sublingual misoprostol in the studies by Unthanan et al. [12] and Abdel-Aal et al. [15] cannot be overlooked. The sublingual route offers rapid absorption and avoids the first-pass metabolism, potentially leading to a more efficient and potent uterine contraction pattern. The strikingly lower cesarean rate of 13.3% with sublingual misoprostol compared to 28.8% with oxytocin in the Unthanan trial [12] is a clinically significant outcome that merits serious consideration. This benefit may be attributed to a more physiological contraction pattern induced by misoprostol or patient selection factors. This finding is consistent with a Cochrane review by Hofmeyr et al. [17] on vaginal misoprostol for labor induction, which also reported a reduction in the rate of cesarean section compared to other induction methods, including oxytocin, in some contexts.

The heterogeneity in efficacy outcomes can be partially explained by variations in study population characteristics and misoprostol administration protocols. The population in Bender et al. [10] was exclusively nulliparous with an unfavorable cervix, a group known for longer labor durations and higher cesarean rates. In such a cohort, the controlled, titratable nature of oxytocin may offer a distinct advantage in managing prolonged labor. In contrast, the studies favoring misoprostol included mixed populations of nulliparous and multiparous women [12,14,15]. The work by Bhatu et al. [14] further nuances this, suggesting that the benefit of misoprostol in shortening labor might be more pronounced in nulliparous women, while outcomes are comparable for multiparous women. This aligns with the physiological understanding that multiparous women generally have a more favorable response to any induction agent. Furthermore, the route of misoprostol administration appears to be a critical factor. The positive results for misoprostol were most pronounced in studies using the sublingual route [12,15], whereas studies using oral [10,11] and vaginal [13] routes showed more mixed or equivalent results. A meta-analysis by Souza et al. [18] specifically comparing routes of misoprostol administration for labor induction found that sublingual and vaginal routes were more effective than oral administration in reducing time to delivery, supporting the pharmacokinetic rationale for our observed differences.

On the critical issue of safety, the collective evidence from this review is overwhelmingly reassuring. The absence of significant differences in major maternal morbidities such as uterine rupture, postpartum hemorrhage, or intraamniotic infection across all six studies [10-15] is a cornerstone finding. This safety profile is paramount, given the historical concerns regarding uterine hyperstimulation with prostaglandins. While Muhmed and Abdelmoaty [13] directly compared uterine tachysystole and found no significant difference between vaginal misoprostol and oxytocin, other studies, like Bender et al. [10] and Unthanan et al. [12], also reported no excess of hyperstimulation-related complications. This suggests that with the dosages and regimens used in these modern trials (e.g., 25μg oral misoprostol [11] or 50μg sublingual [15]), the risk of dangerous uterine overactivity is minimized. This finding corroborates the results of a large prospective cohort study by Dibbs et al. [19], which concluded that low-dose misoprostol protocols are not associated with an increased risk of uterine rupture in term pregnancies compared to oxytocin. Similarly, neonatal outcomes were consistently favorable and equivalent between groups. Parameters such as Apgar scores, NICU admission rates, and fetal distress were no different, reinforcing the conclusions of a comprehensive systematic review by Thomas et al. [20] that found no evidence of increased neonatal harm with misoprostol use for labor induction at term compared to other agents.

An interesting, though less consistent, finding relates to patient satisfaction and minor maternal side effects. Bender et al. [10] reported higher patient satisfaction in the oxytocin group, which may be linked to the shorter labor duration or perhaps a perception of more controlled, hospital-managed care with an intravenous drip. Conversely, Abdel-Aal et al. [15] noted a slightly higher incidence of non-significant maternal side effects like nausea with sublingual misoprostol. This trade-off between efficacy, route of administration, and patient-centered outcomes is an important consideration for clinical practice and shared decision-making. The convenience and non-invasiveness of oral or sublingual misoprostol could be a significant advantage in resource-limited settings or for patients desiring greater mobility during early labor, a factor highlighted in a qualitative study by Paul et al. [21] on women's experiences with induction agents.

When contextualizing these findings, it is essential to consider the methodological rigor of the included evidence. The risk of bias assessment indicated that the majority of the evidence [10-13,15] originates from trials with a low risk of bias, which strengthens the validity of our conclusions. The one study with a high risk of bias [14] was a comparative clinical study, and its findings, while consistent with the overall narrative on safety, should be interpreted with more caution regarding its efficacy claims.

Limitations

This systematic review has several limitations that must be acknowledged. First, the relatively small number of included studies (n=6) and the moderate total sample size (n=806) limit the statistical power for a robust quantitative meta-analysis and the exploration of rare but serious adverse events. Second, significant clinical heterogeneity was present, including variations in the route and dosage of misoprostol, the specific oxytocin protocol used, and differences in patient populations (e.g., parity, cervical status). This heterogeneity precludes definitive, one-size-fits-all conclusions and suggests that the optimal choice of agent may be context-dependent. Third, the exclusion of four studies due to paywall restrictions introduces a potential for publication bias, as we cannot rule out the possibility that these unavailable studies contained findings that could have altered our synthesis. Finally, the applicability of findings from specific contexts, such as a single urban tertiary care center in the USA [10], may be limited when extended to other healthcare settings, especially low-resource environments where misoprostol’s stability and ease of administration offer significant advantages.

## Conclusions

Both misoprostol and oxytocin are effective and safe for labor induction in women with term PROM. The choice between them is not a matter of superiority but rather of tailoring the agent to the clinical context and patient preferences. Oxytocin may be the preferred option in settings that allow for continuous monitoring, especially for nulliparous women with an unfavorable cervix, where achieving a shorter labor duration is a key objective. In contrast, misoprostol, especially via the sublingual route, presents a highly effective alternative that may reduce cesarean section rates and offers logistical advantages, making it particularly valuable in diverse healthcare environments. The favorable safety profiles for both mother and newborn should offer clinicians confidence in using either agent. Future research should focus on large, pragmatic trials that directly compare different misoprostol regimens with standardized oxytocin protocols in well-defined patient subgroups to better guide personalized clinical decision-making.

## Appendices

**Table 4:** Search strategy for ClinicalTrials.gov, PubMed, Embase, and Scopus

Database	Search string
ClinicalTrials.gov	Search Terms: (term prelabor rupture of membranes OR PROM OR premature rupture of membranes) AND (induction of labor OR labour induction) AND (misoprostol OR Cytotec) AND (oxytocin OR Pitocin) Filters: Status: Completed | Study Results: With Results
PubMed	Search Query: (("Fetal Membranes, Premature Rupture"[Mesh] OR "prelabor rupture of membranes" OR "premature rupture of membranes" OR PROM OR "rupture of membranes")) AND ("Labor, Induced"[Mesh] OR "induction of labor" OR "labour induction" OR "cervical ripening")) AND ("Misoprostol"[Mesh] OR misoprostol OR Cytotec) AND ("Oxytocin"[Mesh] OR oxytocin OR Pitocin) Filters Applied Post-Search: Publication date: Last 5 Years | Language: English
Embase (via Elsevier)	Search Query: ('premature rupture of fetus membrane'/exp OR 'prelabor rupture of membranes' OR 'premature rupture of membranes' OR PROM) AND ('labor induction'/exp OR 'induction of labor' OR 'labour induction' OR 'cervical ripening') AND ('misoprostol'/exp OR misoprostol OR Cytotec) AND ('oxytocin'/exp OR oxytocin OR Pitocin) Filters Applied Post-Search: Publication date: Last 5 Years | Language: English
Scopus	Search Query: TITLE-ABS-KEY ( ( "prelabor rupture of membranes" OR "premature rupture of membranes" OR PROM ) ) AND TITLE-ABS-KEY ( ( "induction of labor" OR "labour induction" OR "cervical ripening" ) ) AND TITLE-ABS-KEY ( ( misoprostol OR Cytotec ) ) AND TITLE-ABS-KEY ( ( oxytocin OR Pitocin ) ) Filters Applied Post-Search: PUBYEAR > 2018 (or as needed for 5-year window) | LANGUAGE ( English )
